# Repellent Effect of Volatile Fatty Acids on Lesser Mealworm (*Alphitobius diaperinus*)

**DOI:** 10.3390/insects9010035

**Published:** 2018-03-16

**Authors:** Bartosz Baran, Michał Krzyżowski, Mikołaj Cup, Jakub Janiec, Mateusz Grabowski, Jacek Francikowski

**Affiliations:** 1 Department of Animal Physiology and Ecotoxicology, Faculty of Biology and Environmental Protection, University of Silesia, 40-007 Katowice, Poland; michal.krzyzowski@o2.pl (M.K.); jacek.francikowski@us.edu.pl (J.F.); 2 College of Inter-Faculty Individual Studies in Mathematics and Natural Science, University of Warsaw, 00-927 Warszawa, Poland; mkicup@interia.eu; 3 Faculty of Physics, University of Warsaw, 00-927 Warszawa, Poland; jakub.janiec@me.com; 4 Department of Biophysics, Medical University of Silesia, 41-200 Sosnowiec, Poland; mateusz.grabowski@med.sum.edu.pl

**Keywords:** volatile fatty acids, *Alphitobius diaperinus*, locomotor activity, repellency

## Abstract

Volatile fatty acids (VFAs) are a group of common metabolites and semiochemicals mediating information transfer between higher organisms and bacteria, either from microbiome or external environment. VFAs commonly occur among various insect orders. There are numerous studies exploring their influence on the behavior of different insect species. In relation to the papers published by J. E. McFarlane in 1985, we assessed the effects of formic, acetic, propionic, butyric and valeric acids on the spatial preference of the lesser mealworm (*Alphitobius diaperinus*), a common pest of stored food grain products and the poultry industry. The main aim of the presented study was to provide new angles in VFA research, recreating the classical study both with new methods and on economically significant pest species. This paper presents a novel method of continuous, simultaneous assessment of site preference and the travelled distance in a constant-flow olfactometer. All the tested VFAs, except valeric acid, had a significant repellent effect, with formic acid being effective even at the lowest used concentration. Additionally, the VFAs significantly altered the distance travelled by the insects. The obtained results indicate a potential role for VFAs in the olfactory guided behavior of *A. diaperinus*. It is suspected that the reaction to the presence of VFAs may deviate from the specificity of species’ original habitat.

## 1. Introduction

Volatile fatty acids (VFAs) are carboxylic acids with an aliphatic chain consisting of up to six carbon atoms as produced by the microorganisms, which are either present in the environment or inhabit the animals’ digestive tracts [1]. Over the last decades, VFAs have been extensively investigated due to their potential role as a group of universal infochemicals dedicated to transferring information between higher organisms, native gut microbiome, and their environment. The composition of VFAs among different insect orders is fairly similar. Therefore, studying the role of VFAs may greatly contribute to the general understanding of spatial behavior and chemical ecology of various species [2]. Papers published by McFarlane [2,3,4,5], are important in this field, and as such, directly inspired the presented study. For the purposes of this paper, a flexible, cost-effective system, and a new method of analyzing the influence of volatile compounds on spatial preference and behavioral activity of insects was developed.

Results published by McFarlane [2,6] showed the presence of VFAs in various habitats, body surfaces, and feces of many insect species. Over time, speculation grew about their potential role in the information transfer. Given their characteristics, including high volatility and wide availability in the environment, these compounds present a high potential for information transfer, such as an indication of insect population density in a given area.

VFAs should not be treated as pheromones in the strict sense, as those are considered to be deliberately produced and secreted by an animal, whereas VFA are by-products of digestive processes in the insect’s gut. Therefore, they should be considered in the broader category as infochemicals.

In the presented study, the effects of formic, acetic, propionic, butyric, and valeric acid on the cosmopolitan pest *Alphitobius diaperinus* were investigated. It is well known for its rapidly increasing resistance to pesticides and for being the cause of major economic losses in the poultry industry [7]. The compounds were selected for their reported repellent efficiency against the insect related to *A. diaperinus*—*Tenebrio molitor*—described by Weaver [6,8].

## 2. Materials and Methods

### 2.1. VFAs

Carboxylic acids of increasing carbon chain length (analytical grade purity) used in the presented research—formic, acetic, propionic, butyric and valeric acid—were obtained from POCH S.A.

### 2.2. Insects

In the experiment, specimens of both sexes of *Alphitobius diaperinus* (Panzer) were used. The insects were acquired from infested oatmeal storage, and subsequently reared in constant conditions of 30 ± 1 °C, 50% relative humidity, and photoperiodic regime of 12/12 h light/dark. The culture was maintained on water, ad libitum, and standard dog food pellets (Pedigree).

### 2.3. Behavioral Tests

**Figure 1:**
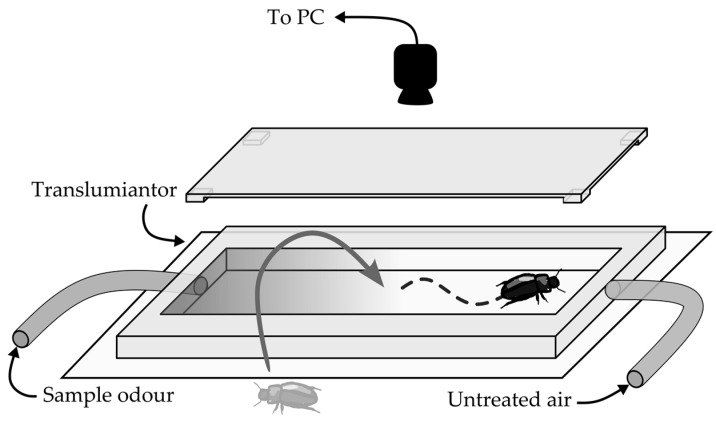
Ideological scheme of experimental setup.

The paradigm applied in the presented research is an iteration of the classic T-maze, in which an insect can choose between two turns of the maze. Each insect was placed separately in the center of a rectangular chamber (3 mm height, 15 mm width, 160 mm long) made of clear Lucite, with silicon tubes attached to both ends (Figure 1). The chamber was closed with a snap-on clear lid with vents along all the edges. To provide sufficient venting, the whole setup was placed in an extensively vented recording container. The tubes provided a constant flow of clean, humidified air from one end, and humidified air with the tested odor from the other one. The airflow was kept at 10 L/h. Inlet air was pumped through a bubbler with mineral oil (to capture potential contaminants from the pump) and was then separated into either a water bubbler or a bubbler with an aqueous solution of carboxylic acid. Constant homogeneous background light was provided with a red transilluminator placed below the setup. The insects were able to explore the chambers freely. The experimental procedure was recorded with Microsoft LifeCam 500 webcam and VirtualDub 1.10.4 software as .avi files. To assess effects of each concentration, 48 insects were used. Each insect was put in the chamber separately, and left undisturbed for 10 min. Then the recording was started. The recordings lasted for 10 min at the frame rate 15 fps, and a resolution of 640 × 860 px. The setup was placed in an enclosed, ventilated test chamber, providing isolation from external visual and acoustic stimuli. For each VFA, a series of dilutions was prepared, namely 0.0001, 0.001, 0.01, 0.1, 1, 10 M in ultrapure, deionized water. The bubbler providing the control airstream was filled with an equal volume of ultrapure water.

### 2.4. Data Analysis

**Figure 2:**
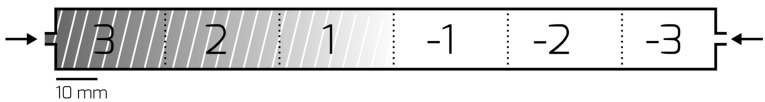
Test chamber divided into compartments, coloration indicates the odor end and the gradient of odor concentration (assessed prior to study with NH_4_Cl deposition).

An analysis of the insects’ movement was performed with SwissTrack^®^ software [9]. The acquired trajectories were analyzed with t R with *adehabitat* package [10]. For the analyses, the chamber was divided into six fictive compartments (Figure 2).

For each compartment, a value (*I_i_*) between −3 (control end—untreated air) to 3 (odor end) was then assigned. Preference index (PI) was calculated by multiplying a compartment value by the time (t) which the insect spent in each specific compartment (frames).

$$\mathrm{PI}=\overset{6}{\underset{i=1}{\sum }}t*I_{i}$$

I={−3,−2,−1,1,2,3}

Results for all the compartments were totaled (min −27,000; max 27,000). When the value of PI was positive, then the tested odor was considered attractant. Consequently, when it was negative it was considered a repellent. Overall characteristics describing movement dynamics were calculated, including the total distance travelled by every insect during the test and their total resting time.

Statistical analyses were conducted with Statistica^®^ software v10. The groups were compared using non-parametric Kruskal–Wallis test (Kruskal–Wallis ANOVA) with the median test. Significance level of 0.05 was applied. For multiple comparisons, Bonferroni correction was used.

## 3. Results

**Figure 3:**
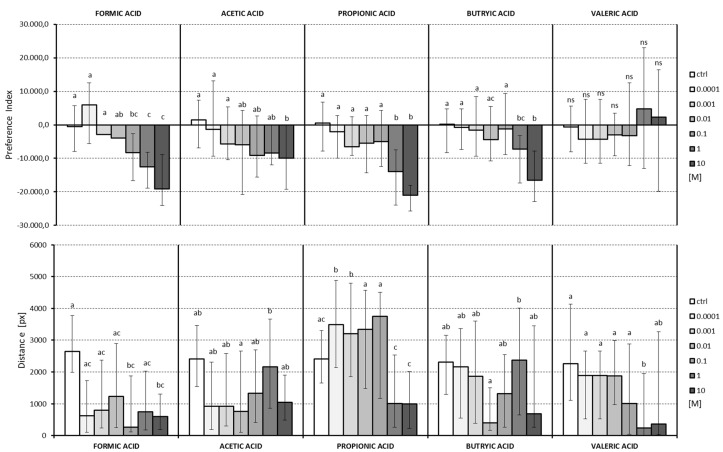
Preference index (PI) and distance travelled by insects exposed to VFAs in different concentrations (M) and control group. Letters indicate statistically different groups, Kruskal–Wallis test, *p* < 0.05.

Analyses of the PI (Figure 3) indicated that all the tested VFAs, which caused statistically significant alteration of behavior, exhibited repellent properties. Formic acid, the shortest carboxylic acid, was the most effective. Just 0.1 M concentration showed a repellent effect, significantly reducing both the insects’ covered distance and the movement time. For acetic acid, the repellent effect was only observed at the highest concentration of 10 M. It slightly affected the distance travelled by the insects, excluding 1 M concentration. Propionic acid had a repellent effect at 1 M and 10 M. An increased in distance travelled, with respect to other groups, was observed in 0.0001 M and 0.001 M groups. It was also possible to observe a decrease in the travelled distance, in relation to other concentrations of 1 M and 10 M. The butyric acid showed a repellent effect at 1 M and 10 M concentrations. All the changes in the distance travelled by the insects, excluding those exposed to the 1 M concentration, were statistically non-significant. Valeric acid did not show any repellent effect at any of the concentrations. The change in the distance travelled was observed only at the concentration of 1 M.

## 4. Discussion

The setup developed for the presented research is an iteration of a T-maze olfactometer. Despite several reports describing similar systems [11,12], the presented solution is based on the open access software, and is assembled from easily available parts. In addition, its construction enables easy adaptation to the conducted experiments. The results obtained in this study indicate the developed system is highly useful as a tool to assess the effects of volatile substances on the behavioral parameters of insect activity.

The findings show statistically significant differences in the studied parameters between different acids and their concentrations. The results are coherent and demonstrate a relation between the acid chain length and its repellent properties, which decreased (with the exception of acetic acid) with the increasing length. The obtained results are different from the ones acquired by Weaver [8] in the study on *Tenebrio molitor*, where acetic acid was reported to be the most effective. In the presented research, formic and propionic acids were the most effective as repellents. Such a difference may be caused by using larvae instead of imagoes. However, the fatty acids may potentially play a role in the spatial preference behavior of *A. diaperinus* and the colony population dynamics. In overcrowded populations, the concentration of the volatile compounds correlates with the increasing number of individuals. The elevated concentration of these substances has a strong repellent effect which, by promoting dispersion of insects, prevents further overcrowding, and therefore modulates the population density by means of a negative feedback loop.

In the natural habitat, *A. diaperinus* occurs at the bottom of the caves inhabited by bats. Guano contains urea [13], which is metabolized by microorganisms into various VFAs [14]. Consequently, the different concentrations of VFAs are commonly present in the natural environment of *A. diaperinus*. Microbiological guano transformations progress in stages [15], hence, it is possible that different profiles and concentrations of VFAs indicate important information about the quality of a potential habitat. In secondary habitats inhabited by *A. diaperinus* (hen houses), VFAs also occur due to bacterial fermentation of uric acid, and may be one of the evolutionary factors which predispose *A. diaperinus* to such quick and efficient colonization of new niches. In coherence with the literature, high concentrations exhibit the repellent effect which indicates unsuitable habitat. Moreover, the attractant effect may occur in response to the mix of VFAs and other substances [16].

VFAs are compounds closely related to the activity of microorganisms and the communication between organisms and microbes. However, McFarlane’s research, as well as the presented work, indicates a possibly wider role of VFAs as universal information factors, including the transmission of information in the external environment. The reputed properties of some VFAs give hope for the development of novel, information-based methods of fighting and deterring *A. diaperinus*, which may help to significantly reduce use of synthetic pesticides.
